# *Ascophyllum nodosum* SWE enhances root anatomy, but not POD activity in both a salt-tolerant and salt-sensitive soybean ( *Glycine max)* variety exposed to salt stress

**DOI:** 10.17912/micropub.biology.001046

**Published:** 2024-03-22

**Authors:** Elena Hoang, Paul Stephenson

**Affiliations:** 1 Biology, Rollins College, Winter Park, Florida, United States; 2 Biology, Rollins College

## Abstract

There is growing evidence that seaweed extracts (SWE) may be a solution for mitigating the negative effects of salt stress on crop yield and quality, as they introduce bioactive ingredients able to regulate the expression of growth-inducing and stress-responsive genes. We demonstrate that SWE slightly ameliorated the negative physical growth effects of salt stress, especially in the root anatomy of the salt-sensitive (Clark) variety. The SWE did not stimulate or enhance peroxidase (POD) activity in either the salt-sensitive (Clark) or salt-tolerant variety (Manokin). However, a complete assessment of other antioxidant enzymes (SOD, CAT, APX) involved in the ROS detoxification process is further required.

**Figure 1. Salt stress impacts on root volume are alleviated by treatment with biostimulant seaweed extract (SWE), and increased peroxidase activity is observed in salt tolerant soybeans at 0.1 M NaCl (-SWE): A-B):**
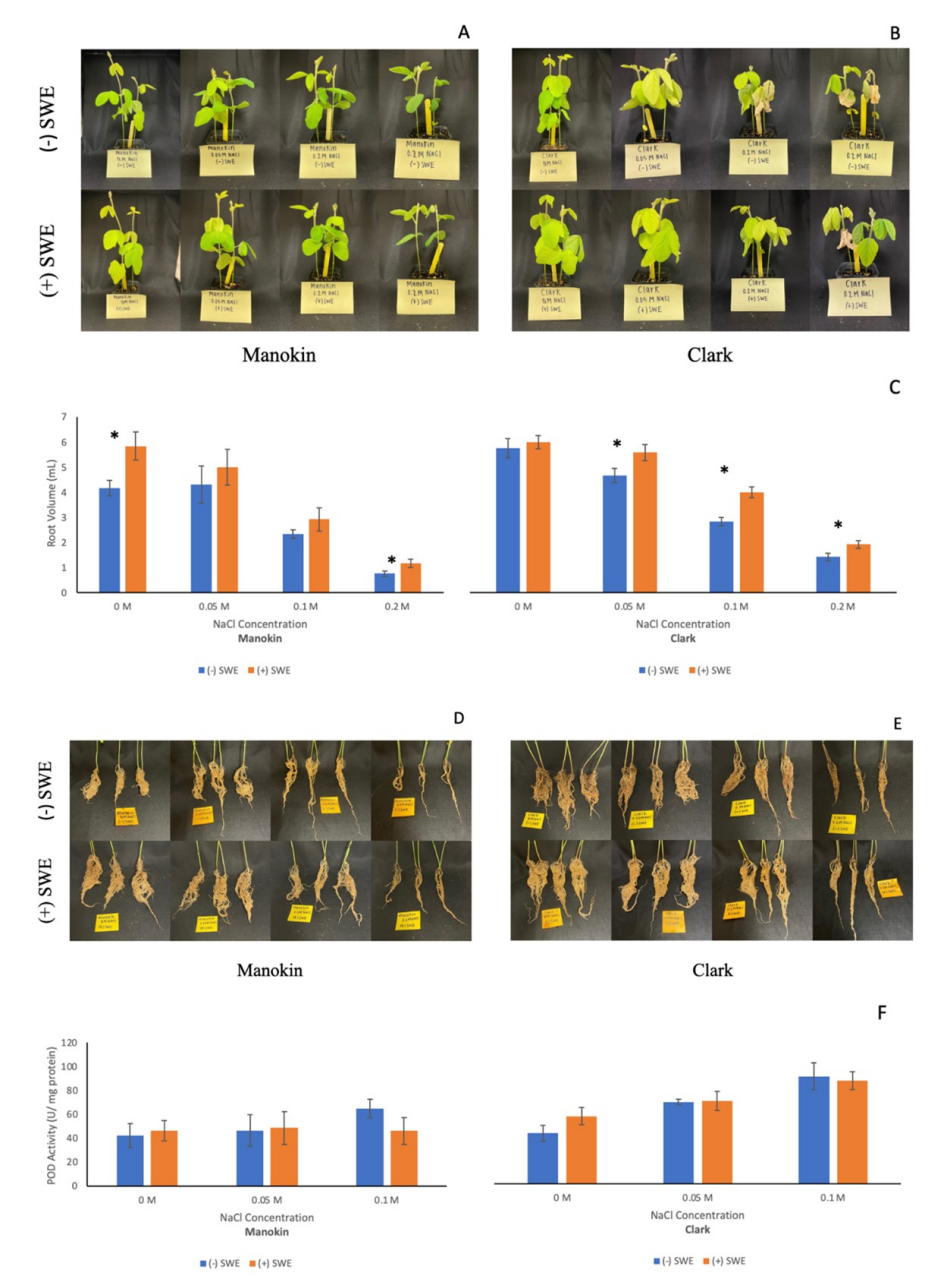
Images of salt tolerant Manokin and salt sensitive Clark individuals at the end of the growth period across all salinity levels. **C)** Average root volume of Manokin and Clark seedlings for each treatment. (+) SWE replicates were foliar sprayed with 10 mL of Maxicrop Liquid Seaweed solution each week and volume was measured by water displacement. * indicates that a T-test yielded a significant difference (p < 0.05) between the means. Error bars represent standard error. **D-E)** Images of salt tolerant Manokin and salt sensitive Clark root mass at the end of the growth period across all salinity levels. **F)** Average peroxidase activity in Manokin and Clark seedlings for each treatment. (+) SWE replicates were foliar sprayed with 10 mL of Maxicrop Liquid Seaweed solution each week. Peroxidase activity was measured after 30 days using leaf tissue samples (* indicates that a T-test yielded a significant difference (p < 0.05) between the means). Error bars represent standard error.

## Description

Soil salinity, a climate change related abiotic stressor, disrupts the normal growth, development, and metabolic function of sensitive plants (Rai et al., 2021) . While necessary micronutrients for plants, both Na ^+^ and Cl ^-^ can easily be accumulated to toxic levels. The accumulation of Na ^+^ in foliar tissue can competitively inhibit the activation of stress responses, as well as lead to decreasing levels of photosynthesis and eventual chlorosis linked to reduction of chlorophyll content (Munns and Tester, 2008; Kibria and Hoque, 2019; Wu and Li, 2019) . Chloride ions are normally essential to plants for their role in regulating osmotic potential and the turgor pressure of cells, but in excessively high concentrations, Cl ^-^ can induce osmotic stress by creating an imbalance in the salt concentration gradient between plant cells and the soil (Zhao et al., 2021) . Plants will respond to the water loss by rapidly inducing stomatal closure (Zhao et al., 2021) , which leads to the mass production of reactive oxygen species (ROS) that are characteristic of secondary oxidative stress (Abogadallah, 2010; Das and Roychoudhury, 2014) .

In low concentrations and under normal, unstressed conditions, ROS are signaling molecules that participate in various biological processes (Zhao et al., 2021) . However, the accumulation of ROS during salt stress conditions causes oxidative damage in plant cells through lipid peroxidation and protein or nucleic acid denaturation (Abogadallah, 2010) . H _2_ O _2_ is particularly noteworthy for its long half-life and ability to diffuse across membranes, enabling it to disrupt the activity of Calvin-cycle related enzymes (Das and Roychoudhury, 2014) . In plants, antioxidant regulation can eliminate ROS through a system of scavenger enzymes, with each antioxidant enzyme performing its own specialized function. Superoxide dismutase (SOD) primarily functions as the first defense against oxidative stress, as it converts the ROS O ^•-^_2_ into another ROS form, H _2_ O _2_ , which is then detoxified by any of the three enzymes: catalase (CAT), ascorbate peroxidase (APX), or peroxidase (POD) (Abogadallah, 2010) . The peroxidases, with their high affinity for H _2_ O _2_ , exhibit more efficient and fine regulatory scavenging activity with their decomposition of the ROS (Das and Roychoudhury, 2014) .

Previous studies on both crop and wild species have demonstrated that an increase in the expression of these scavenging enzymes can improve plant tolerance to soil salinity and oxidative stress (Abogadallah, 2010; Das and Roychoudhury, 2014) , suggesting the presence of a link between tolerance to saline conditions and the increased expression of genes regulating the antioxidant enzymatic system involved in ROS detoxification. While soybeans ( *Glycine max* ) are classified as being a moderately salt sensitive crops (Munns and Tester, 2008) , multiple salt-responsive, or stress-responsive genes, that are capable of conferring salt tolerance have been identified in several lines (Munns and Tester, 2008; Guan et al. 2014; Jin et al., 2019; Wu and Li, 2019; Guo et al., 2021) . Studies regarding methods for reducing damage to crop yields have demonstrated that seaweed extracts (SWE) used as biostumulants are able to stimulate adaptive defense in plants against abiotic factors by influencing changes in the expression of relevant stress-response genes (Shukla et al., 2018; Langowski et al., 2021) . Seaweed extracts, when applied to plants, have consistently resulted in positive benefits such as increased root length, leaf surface area and shoot length, chlorophyll content and stomatal conductance, phytohormones, and fruit quality (Boukari et al., 2020; Ali et al., 2021). However, the specific effects and exact mechanisms by which these benefits occur are dependent on the extract species, the extraction method, and the species to which it is being applied (Shukla et al., 2019; Boukari et al., 2020; Ali et al., 2021; Bahmani Jafarlou et al., 2022) . This study utilized the commercially produced biostimulant Maxicop (Ohrstrom’s Maxicrop, c2023) derived from the brown seaweed *Ascophyllum* *nodosum* . Unlike traditional fertilizers that serve to provide essential nutrients like nitrogen and phosphorus (Rai et al., 2021) , Maxicrop has a 0-0-1 ratio of Nitrogen-Phosphorus-Potassium (Ohrstrom’s Maxicrop, c2023). Instead, SWE extracts are enriched in polysaccharides, phenolic acids, and phytohormones, that are able to stimulate growth and productivity processes in plants to which they are applied (Khan et al., 2008; Shukla et al., 2018; Boukari et al., 2020; Ali et al., 2021) . This study aimed to assess the effectiveness of an *Ascophyllum nodosum* derived SWE in mitigating negative salt stress effects by measuring physical growth and using antioxidant enzyme activity as an indicator of oxidative stress. To compare how the biostimulant effects may vary based upon genetic differences, we utilized a two-factor experimental design performed simultaneously for both a salt-tolerant (Manokin) and salt-sensitive (Clark) soybean cultivar. The replicates were grown under a range of saline conditions (0.05 M NaCl, 0.1 M NaCl, 0.2 M NaCl, with water as a control), and either treated [(+) SWE] or untreated [(-) SWE] with weekly foliar applications of SWE.

At the end of the 30-day growth period, the salt-tolerant Manokin seedings did not display the same physical symptoms of chlorosis and necrosis ( Figure 1A ) that were visible amongst the salt-sensitive Clark seedlings ( Figure 1B ). For both varieties under both experimental and control conditions, root volume greatly decreased as salinity increased, but (+) SWE seedlings exhibited greater root volume than (-) SWE seedlings at the same salinity level ( Figure 1C ). Without interaction and independent of one another, both salinity and seaweed extract applications were found to have a significant effect on root volume. SWE treatments appeared to be most effective at enhancing root growth in the salt-sensitive Clark variety, as the mass of root material for Manokin seedlings was observed to grow visibly shorter and thinner as salinity increased ( Figure 1D ), while salt-sensitive Clark seedlings visually maintained a greater root volume, even for individuals exposed to 0.2 M NaCl ( Figure 1E ).

In order to assess the impact of SWE treatment on the ROS scavenging system, we measured peroxidase (POD) activity in leaf tissues samples. For the salt tolerant Manokin seedlings, neither salinity nor seaweed extract applications revealed a significant impact on POD activity. The (+) SWE Manokin seedlings exhibited consistent, unchanging POD activity levels across all tested salinity levels ( Figure 1F ). No change was observed in the average POD activity of (-) SWE replicates from controls and samples treated with 0.05 M NaCl, but (-) SWE seedlings treated with 0.1 M NaCl displayed increased activity ( Figure 1F ). In contrast, salinity, independently and without interaction, did significantly impact peroxidase activity levels for Clark seedlings. As salinity increased, the peroxidase activity of Clark seedlings also increased, but there was no significant difference between (-) SWE and (+) SWE replicates ( Figure 1F ).

While we did not see a change in POD activity for the Clark seedlings treated with SWE, we did observe a significant enhancement in root volume. As this trend was not reflected in the salt-tolerant Manokin variety, we propose that these results are related to the lack of a functional salt-responsive gene for the SWE to act upon, leading the sensitive variety to respond to salt stress through morphological adaptations. While it is generally recognized that the influx of sodium ions in roots results in a reduction of root elongation, epidermis thickness, and architecture, some studies have shown occasional enhancements to root anatomy when exposed to saline conditions similar to those used in our study (0.05 M and 0.1 M NaCl) (Hasanuzzaman et al., 2022) . The phenotypic plasticity of roots allows some crop species, like soybeans, to maintain stability and better exploit resources in the soil by altering growth in response to various stressors (Muller et al., 2021), and a previous study characterizing root over-proliferation has suggested that the behavior is genotype, or context-dependent (Chen et al., 2021) . These factors could explain the phenomenon of stimulated root growth in (+) SWE Clark seedlings under salt stress.

Although we did not see an increase in POD activity for (+) SWE Manokin seedlings, our original hypothesis that the biostimulant can upregulate antioxidant enzyme activity cannot be entirely excluded. A separate study assessing antioxidant activity in other soybean cultivars found that a salt tolerant variety exhibited higher levels of SOD and APX activity in the roots and leaves than a salt sensitive variety (Yu and Liu, 2003) . This suggests that, although POD activity did not increase, other antioxidant enzymes may be upregulated. In order to determine whether seaweed extracts can increase abiotic stress tolerance through enhancing the function of other scavenging enzymes, future research should perform a complete assessment of SOD, CAT, and APX involvement in ROS scavenging. This may help discern differences between the effects on tolerant and sensitive varieties treated with SWE. Despite this, our study still reflects positive benefits associated with seaweed extract applications and crop yield, indicating that there is a generalized mitigation of negative salt stress effects that corresponds to SWE applications, and that genetic differences between cultivars of the same species plays a large role in the appearance of these benefits.

## Methods

***Soybean Growth and Experimental Treatments***

A two-factor experimental design, simultaneously performed for two different soybean cultivars, was utilized to subject soybean seedlings to seaweed extract treatments and different levels of salinity. Seeds of the salt-tolerant Manokin variety (Accession: PI 559932), and the salt-tolerant Clark variety (Accession: PI 548533), were obtained from the U.S. National Plant Germplasm System. In a 4”x4” pot, two seeds of the same variety were planted in pre-moistened Sungro Professional Potting Mix. Three replicate pots per treatment were planted. The replicates were grown under a range of saline conditions (0.05 M NaCl, 0.1 M NaCl, and 0.2 M NaCl, with water as a control), and either treated [(+) SWE] or untreated [(-) SWE] with weekly foliar applications of SWE. Manokin seedlings and Clark seedlings were grown on separate shelves within the same Conviron Adaptis Plant Growth Chamber. A scheduled cycle of light, humidity, and temperature conditions was controlled by a CMP6010 Control System (Table 1). Seaweed extract treatments were prepared by diluting Ohstrom’s Maxicrop Liquid Seaweed, a commercially available product derived from *Ascophyllum nodosum* (Ohrstrom’s Maxicrop, c2023), in a ratio of one ounce to one gallon of water per the manufacturer’s instruction. The solution had a 0-0-1 ratio of Nitrogen-Phosphorus-Potassium. SWE treatments began on the day of planting, with continued applications at 1-week intervals. The (+) SWE seedings were uniformly foliar sprayed with 10 mL of the prepared Maxicrop solution, while the (-) SWE control seedlings were treated with 10 mL of ddH _2_ O.

**Table 1. The scheduled settings for temperature, humidity, and light intensity in the Conviron Adaptis Plant Growth Chamber.** Conditions were controlled by a CMP6010 Control System. The observed average relative humidity ranged between 60-80%. A photometer measured Light Level 1 to be an intensity of 62.4 mm/sec m ^2^ per uA, and Light Level 2 to be 128.0 mm/sec m ^2^ per uA.

**Table:** 

**Time**	**Temperature**	**Relative Humidity**	**Light Level**
00:00	21.0 °C	45%	0
07:00	24.0 °C	45%	1
14:00	26.0 °C	45%	2
22:00	24.0 °C	0%	0

***Physical Measurements***

At the end of the 30-day growth period, there were 6 replicates per treatment, with the exception of “Manokin, 0.05 M NaCl, (+) SWE”, which yielded only 5 replicates due to an unsuccessful germination. Roots were washed to remove soil and blotted to remove excess water. Photographs of each replicate pot’s root mass were captured using a shadowbox photo studio. Root volume was estimated through water displacement by the introduction of roots into a graduated cylinder. To account for roots tangled between two seedlings, the volume per replicate pot was measured and used to calculate the average root volume per individual plant.

***Peroxidase Activity Assessments***

Peroxidase assays were not performed on samples of either variety treated with 0.2 M NaCl due to an insufficient collection of leaf material for all replicates. A total of 6 replicates was assayed for each treatment, with the exception of “Manokin, 0.05 M NaCl, (+) SWE”, which only produced 5 replicates due to an unsuccessful germination. Samples from each replicate were removed from storage at -80 °C and prepared before assaying. Per a 9:1 ratio of volume extraction buffer to weight of tissue (g), a 0.200 g sample of leaf tissue was homogenized in 1.8 mL of 10 mM PBS (pH 7.4) and centrifuged at 10,000 g for 10 minutes at 4 °C. The supernatant was preserved on ice for use as enzyme extract. Samples were further diluted with 10 mM PBS (pH 7.4) at a dilution factor of 1:2 before the protein concentration was measured and peroxidase activity was evaluated. Protein concentration in mg/mL was determined by the Bradford method (Bradford, 1976) , using bovine serum albumin (BSA) in 10 mM PBS (pH 7.4) for the protein standards. The peroxidase activity of each sample was determined according to manufacturer’s protocols using the Peroxidase (POD) Activity Assay Kit for Plant Samples (Catalog No: E-BC-K227-S) from Elabscience.

***Statistical Analysis***

All data was analyzed using two-way ANOVA using MATLAB software. In order to accommodate for the unbalanced sample sizes of the Manokin replicates, an N-way ANOVA analysis of variance was utilized. The means among treatments were compared using T-tests after calculating homogeneity of variance. An ANOVA or t-test P value ≤ 0.05 was considered statistically significant.
